# Additive manufacturing technologies in the oral implant clinic: A review of current applications and progress

**DOI:** 10.3389/fbioe.2023.1100155

**Published:** 2023-01-20

**Authors:** Shitou Huang, Hongbo Wei, Dehua Li

**Affiliations:** State Key Laboratory of Military Stomatology, National Clinical Research Center for Oral Diseases, Shaanxi Engineering Research Center for Dental Materials and Advanced Manufacture, Department of Oral Implants, School of Stomatology, The Fourth Military Medical University, Xi’an, Shaanxi, China

**Keywords:** additive manufacturing technologies, 3D printing, oral implantology, surgical guides, customized titanium meshes, dental implants, custom trays, implant models

## Abstract

Additive manufacturing (AM) technologies can enable the direct fabrication of customized physical objects with complex shapes, based on computer-aided design models. This technology is changing the digital manufacturing industry and has become a subject of considerable interest in digital implant dentistry. Personalized dentistry implant treatments for individual patients can be achieved through Additive manufacturing. Herein, we review the applications of Additive manufacturing technologies in oral implantology, including implant surgery, and implant and restoration products, such as surgical guides for implantation, custom titanium meshes for bone augmentation, personalized or non-personalized dental implants, custom trays, implant casts, and implant-support frameworks, among others. In addition, this review also focuses on Additive manufacturing technologies commonly used in oral implantology. Stereolithography, digital light processing, and fused deposition modeling are often used to construct surgical guides and implant casts, whereas direct metal laser sintering, selective laser melting, and electron beam melting can be applied to fabricate dental implants, personalized titanium meshes, and denture frameworks. Moreover, it is sometimes required to combine Additive manufacturing technology with milling and other cutting and finishing techniques to ensure that the product is suitable for its final application.

## 1 Introduction

In recent years, digital technologies, such as computer-aided design/computer-aided manufacture (CAD/CAM), digital intraoral scanners, and additive manufacturing (AM) technologies, have been successfully applied in implant dentistry (Dawood et al., 2015), providing new clinical and technical methods for surgical oral implant operations and restoration manufacture. Digitalization shows a steady development trend in the field of dentistry. As a typical digital manufacturing technology, AM can connect disease diagnosis, treatment planning, and production processes through data flow, forming a fully digital process for dental product processing (Salmi, 2021). Digital workflows not only greatly improve the safety of implant placement and the convenience of manufacturing restoration, but also reduce labor intensity for dentists and provide a satisfactory medical experience for patients (Barazanchi et al., 2017).

Additive manufacturing (AM) technologies, also known as three-dimensional (3D) printing technologies, are rapid prototyping technologies that have gradually become alternative methods for generating products from CAD files in dentistry (Abduo et al., 2014). Previously, numerical control processing technology (NC technology, also known as subtractive machining technology) was typically used. In the industry, NC refers to the use of turning, milling, grinding, and other approaches to remove material from a specific solid object to form a desired shape. As the processed objects in stomatology NC machining are specific oral materials, milling and grinding techniques are often adopted according to the characteristics of the material used and precision manufacturing requirements (Abduo et al., 2014). NC is a mature processing technology that achieves high precision using a wide range of materials and can be used to directly process almost all commonly used stomatological materials. In addition, NC is the first choice for batch fabrication (Petrick and Simpson, 2015); however, the disadvantages of NC include the material waste associated with this technology, which leads to high production costs. Moreover, particularly complex dental auxiliary therapy devices (such as root-analogue implants, personalized titanium meshes, etc.) are difficult to achieve using NC (Dawood et al., 2015).

AM is a processing technology based on discrete stacking forms. The underlying principle involves transformation of a 3D digital model into continuous superposition of a two-dimensional sheet model through a discrete process, where a computer program controls the stacking of materials in order, layer by layer. The most remarkable features of AM are that it overcomes the limitations of subtractive machining technology and can be used to mass produce a variety of products with complex morphology in a short time. In addition, in principle, AM consumes a minimum amount of raw materials, and unformed raw materials (resin liquid, metal powder, etc.) can be reused, which greatly reduces costs (Kessler et al., 2020; Tian et al., 2021).

Overall, these two manufacturing methods (NC and AM) each have advantages and disadvantages, and AM is not conclusively superior to subtractive manufacturing; in most cases, the techniques present complementary advantages (Alammar et al., 2022). In implant dentistry, applications of and research into AM are becoming increasingly extensive, ranging from fabrication of surgical guides and dental implants to dental casts and implant frameworks, among other items. The use of AM has led to a progression of implant dentistry applications from traditional, purely empirical methods to more accurate digital medicine (Schweiger et al., 2021; Huang et al., 2022).

Several reviews on the applications of AM in dentistry have been published (Barazanchi et al., 2017; Revilla-Leon and Ozcan, 2019; Tian et al., 2021); however, few have focused on the application of this technology in oral implantology. Moreover, the materials reviewed have been limited to polymers, and the types of applications covered were not comprehensive (Revilla-León et al., 2020). In this review, we discuss the AM technologies commonly used in oral implantology and their applications. In addition, we compare the accuracy of different AM technologies and describe the clinical applications of products fabricated by AM.

The aim of this review is to provide readers with information on recent progress in the application of AM in oral implantology. As a result, we focus on the following questions: Which AM technologies are commonly used in implant dentistry? What are the applications of AM technologies in implant dentistry? What are the benefits of the application of AM technologies in implant dentistry?

## 2 Additive manufacturing technologies commonly used in implant dentistry

AM refers to a class of manufacturing processes in which parts are built by stacking layers of material on one another. There are seven 3D printing categories in the American Society for Testing and Materials classification standard: vat photopolymerization (VPP), powder bed fusion (PBF), material jetting, binder jetting, material extrusion (MEX), sheet lamination, and directed energy deposition. Although there are various AM processes, not all of them are used in implant dentistry. Technologies frequently adopted in implant dentistry practice include VPP [stereolithography (SLA), digital light processing (DLP), etc.], PBF [selective laser melting (SLM), selective laser sintering (SLS), etc.], and MEX (fused deposition modeling, etc.) (Schweiger et al., 2021). Parts are built directly from digital 3D models created using CAD software. CAD models are converted into many thin layers, and the fabrication facility uses this geometric data to build each layer in turn until the part is completed (Bozkurt and Karayel, 2021). Given this approach, AM is often referred to as layered manufacturing, direct digital manufacturing, or physical free-form manufacturing (Turkyilmaz and Wilkins, 2021).

**FIGURE 1:**
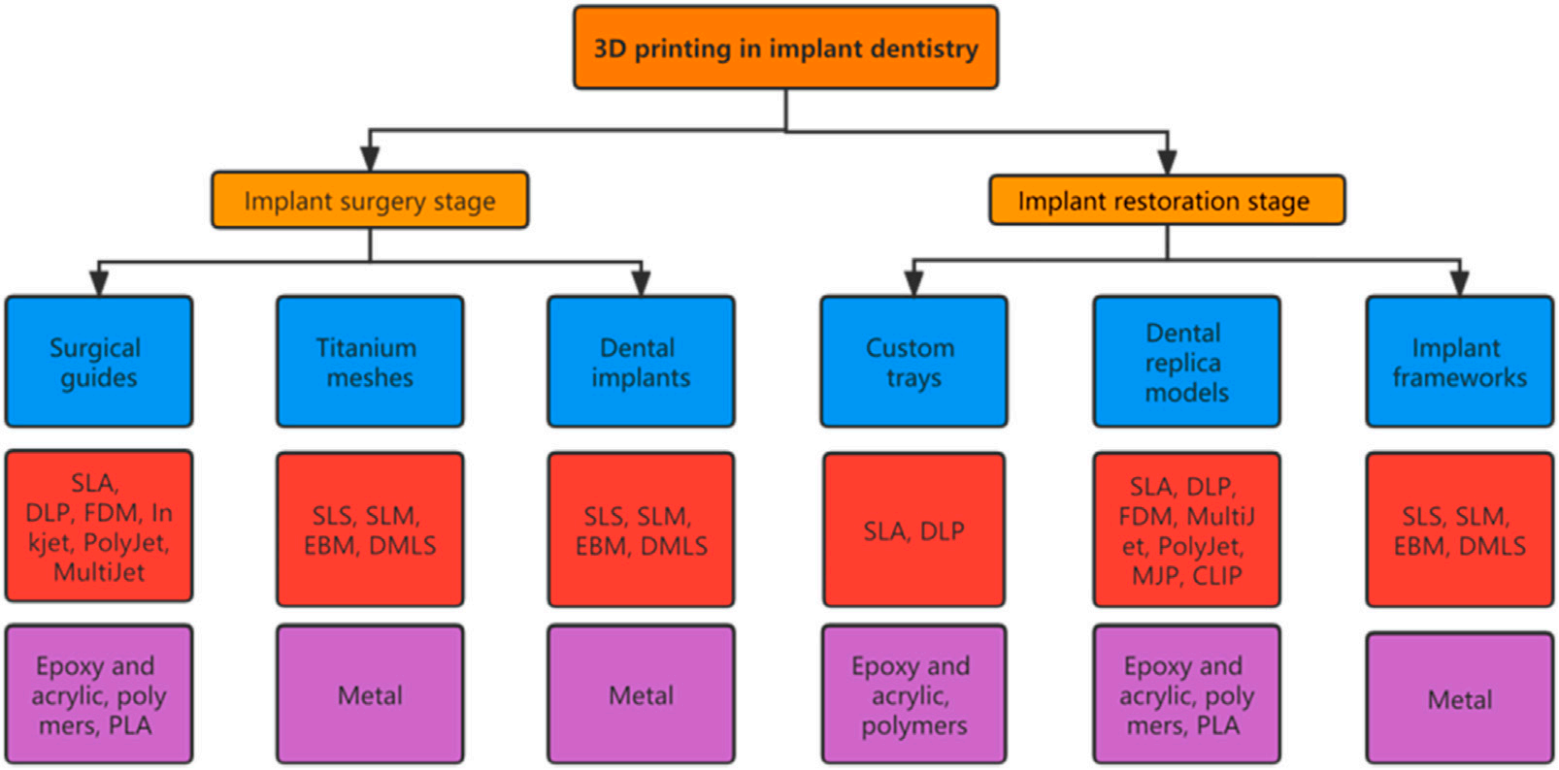
Applications of 3D printing technologies commonly used in implant dentistry. Chart showing the applications (blue boxes), primary 3D printing technologies (red boxes), and materials needed (purple boxes) (Dawood et al., 2015; Khorsandi et al., 2021; Tian et al., 2021).

Each 3D printing technology is based on the principle of the “additive” method, with the main differences among them being the molding methods and materials used. Appropriate 3D printing technologies should be selected according to the application purpose (Figure 1) (Jawahar and Maragathavalli, 2019; Schweiger et al., 2021).

### 2.1 Vat photopolymerization

#### 2.1.1 Stereolithography

SLA is a well-known 3D printing technology that has been used for almost 30 years (Turkyilmaz and Wilkins, 2021), and is primarily applied to print surgical guides, dental model replicas, custom trays, and provisional restorations (Anadioti et al., 2018; Khorsandi et al., 2021; Schweiger et al., 2021). SLA uses a high-intensity ultraviolet light source, which applies the wavelength and heat of light to polymerize and selectively solidify liquid resin for lamination. In this process, to better integrate new layers with previous layers, the polymerization reaction of each layer is usually not fully completed under the direct light source, but further light processing is rather performed after the printing is completed (Pagac et al., 2021). Finally, the support structures added automatically by the printer require manual removal (Revilla-León et al., 2020).

#### 2.1.2 Digital light processing

DLP, as a VPP technology, has attracted wide attention. A major area of DLP printer application is the digital manufacture of dental models (Park et al., 2021). Differences between DLP and SLA include the type of light source and the way the light source is controlled to selectively illuminate and cure the resin. In SLA, the light source is a laser, while DLP uses a projector, similar to a movie projection device, that illuminates the entire shape of the printed object at the surface of the liquid (Pagac et al., 2021; Son et al., 2021). In theory, DLP printing of an object takes less time, because each layer does not require a step-by-step laser scan; however, most DLP devices do not have the high resolution that SLA laser beams can provide (Revilla-León et al., 2020). Therefore, DLP is advantageous for rapid printing of larger parts with fewer details, while SLA is superior for printing accurate parts with intricate details (Huang et al., 2022).

#### 2.1.3 Continuous liquid interface production (CLIP)

CLIP is a variant of DLP considered to be a vat-polymerization technology (Alammar et al., 2022), conducted using a liquid resin cylinder, a bottom-up construction platform, an ultraviolet lamp, an oxygen-permeable window, and a projector. The projector displays a continuous, extremely thin cross section of an object, using ultraviolet light from below (Pagac et al., 2021). Ultraviolet rays harden the liquid in cross section in a cylinder of liquid resin. Simultaneously, a lift pulls the formed objects out of the resin tank (Alammar et al., 2022). The key to CLIP printing is the presence of a window for oxygen and ultraviolet light to pass through at the bottom of the resin cylinder. As oxygen hinders the curing process, resin at the bottom of the cylinder continuously forms a “dead zone” that does not cure. This “dead zone” is very thin, such that ultraviolet light can pass through and solidify the resin above, which does not come into contact with oxygen. As there is no resin stuck to the bottom of the cylinder, the printing speed is very fast, because printing does not occur in the air but in the resin (printing in the air reduces curing speed, due to the presence of oxygen) (Alammar et al., 2022).

### 2.2 Powder bed fusion

PBF is the metal-printing technology most commonly used in dentistry. Ti and Cr-Co alloys are preferred metal materials in biomedical applications, primarily because of their mechanical properties, biocompatibility, thermal, magnetic, and electrical conductivity, and general high temperature resistance (Revilla-León et al., 2017). PBF includes SLS, direct metal laser sintering (DMLS), SLM, and electron beam melting (EBM). These techniques typically use high-powered lasers or electron beams to melt small particles, such as plastics, metals, ceramics, or glass, in powder form (Velasquez-Garcia and Kornbluth, 2021). The powder is usually preheated to a temperature below the melting point of the material before printing begins. The energy source is then controlled by the printer, enabling it to selectively melt powder on the surface of the powder bed. After melting one layer, the powder bed reduces by the height of one layer, and a new powder layer is then laid on top with a roller, subsequently completing printing of the new layer (Revilla-Leon et al., 2019a).

Most non-metallic materials printed by PBF do not require support structures, because the model is always fully wrapped and supported by green powder; however, metallic materials may require support structures to assist in rapidly transferring heat away from the part while reducing expansion during printing. PBF printers can build three-dimensional geometries, such as fine lattices, which are valuable for making prostheses to promote bone ingrowth; hence, PBF technology is widely used in medical fields, including orthopedics and for dental implants, among other applications (Mangano et al., 2014a). In addition, personalized titanium mesh and implant frameworks can be fabricated using this technology (Sumida et al., 2015; Barbin et al., 2020).

The main speed-limiting steps of PBF are the thermal cycle of the machine and post-processing of parts (Attarilar et al., 2021). Most PBF machines need to be warmed up to a certain temperature to start printing, and after printing, cooling is required before the printed parts can be removed from the machine. The post-processing procedures required are also highly dependent on the type of technology and materials used. For example, metal materials require processes such as thermal hardening and residual stress relaxation (Sing et al., 2016). Metal-printed parts may also require subsequent milling with a computer numerical control (CNC) lathe, to achieve a smooth finish, after they have been removed from the printer platform (Bordin et al., 2017).

At present, in the PBF category, SLM makes comprehensive use of cutting-edge technologies, such as new material, laser, and computer technologies, which have great potential for further future development (Ansari et al., 2019). Dental implants printed by SLM have higher density and strength, as well as sufficient dimensional accuracy (Chen et al., 2014), which can be attributed to the SLM forming principle. SLS binds metal or non-metallic powders with high melting points by melting a metal or binder with a low melting point (Jawahar and Maragathavalli, 2019), while SLM uses a high power laser with a small spot to melt metal powder rapidly and completely, and requires higher laser power density than that used for SLS (Revilla-León et al., 2017). In addition, although SLM parts have good mechanical strength, they may have high internal stresses caused by the thermal gradients induced during processing, necessitating additional heat treatment.

EBM is also a powder bed melting technology. The principles of fabrication using EBM and SLM are similar, but the heat sources differ (Velasquez-Garcia and Kornbluth, 2021). Compared with technologies that use lasers as the energy source, EBM has many advantages, such as high energy utilization, no reflection, high power density, and convenient focusing, which can be used to make implants (Raheem et al., 2021).

### 2.3 Material extrusion

The MEX process is also termed fused filament fabrication (FFF) (Bozkurt and Karayel, 2021). The MEX family process, fused deposition modeling (FDM), is the most common type of printing used in medical or dental equipment; however, its printing resolution is inferior to that of SLA (Huang et al., 2017; Oberoi et al., 2018). FDM can handle a variety of materials, such as foundry wax, polyamide (commonly known as nylon), acrylonitrile butadiene styrene plastic, polylactic acid (PLA) plastic, low melting point metals, and ceramics (Lin et al., 2019). In FDM, the thermoplastic material is heated, melted, and extruded uniformly from a nozzle to generate filaments. Simultaneously, the nozzle moves along a specific path, operated by an NC system according to the continuous thin layer data planned by the slicing software, for filling. After cooling, the filamentous material is bonded layer by layer to form a thin cross-section, and finally the layers are superimposed to form a three-dimensional entity (Torabi et al., 2015).

### 2.4 Material jetting

In 2000, Objet (Israel) applied for a patent for PolyJet technology (now owned by Stratasys). PolyJet sprays a liquid photopolymer layer onto the construction tray and immediately solidifies it using ultraviolet light. Compared with SLA, the PolyJet laser spot diameter is 0.06–0.10 mm, allowing much higher printing accuracy than that achieved by SLA, and facilitating fabrication of smooth and precision parts. In addition, due to its high-speed raster construction process, PolyJet can achieve fast printing, and does not require secondary curing. It has been demonstrated that implant guides made using PolyJet are more accurate than those fabricated using SLA technology (Tappa and Jammalamadaka, 2018; Patpatiya et al., 2022).

## 3 Research status on additive manufacturing applications in implant dentistry

**FIGURE 2:**
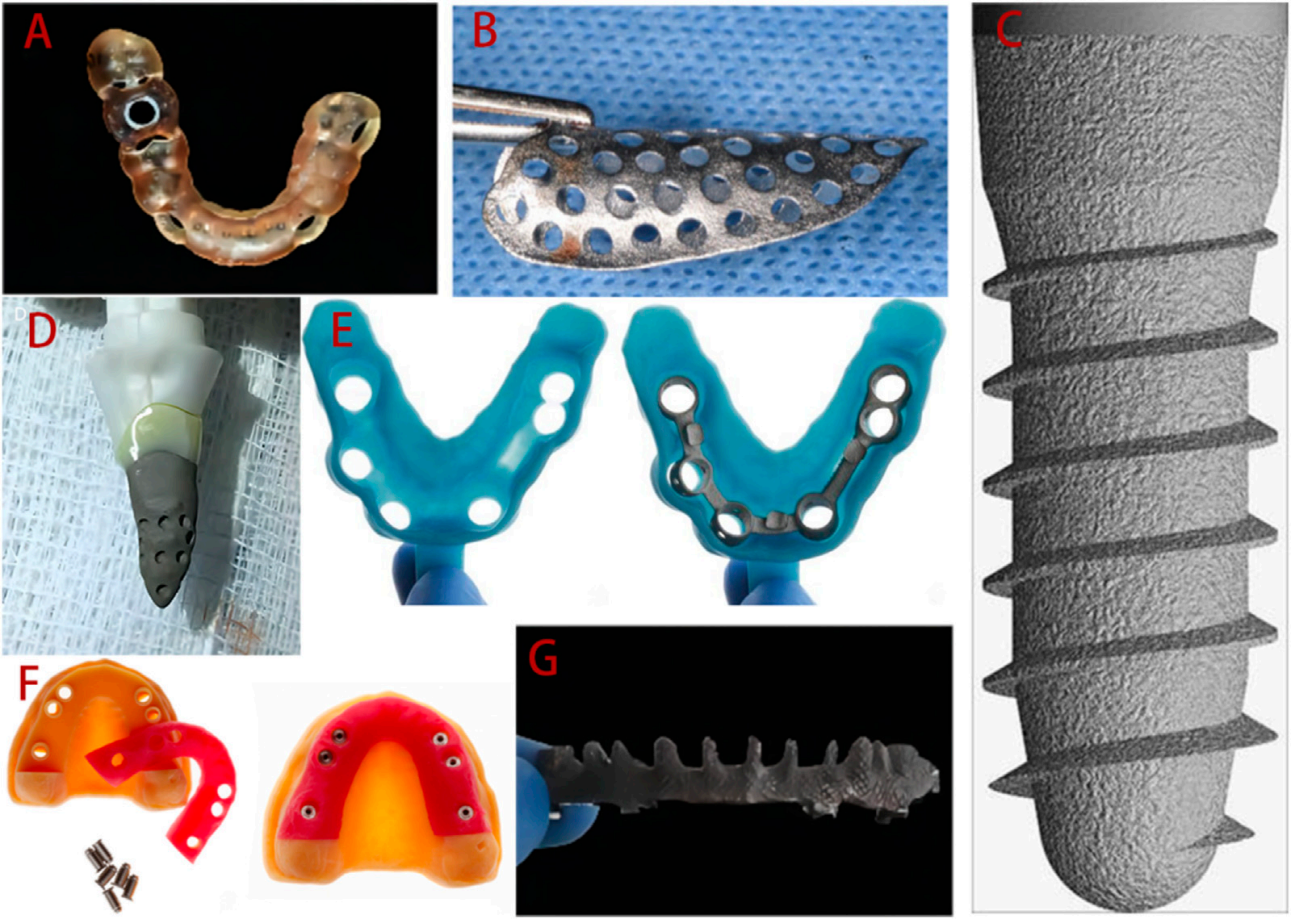
Applications of 3D printing technologies in implant dentistry clinical practice. **(A)** 3D printed surgical guide. **(B)** Personalized titanium mesh. **(C)** Standard implant. **(D)** Customized root-analogue implant. **(E)** Custom tray. **(F)** Implant models. **(G)** Implant framework. Adopted from (Mangano et al., 2012b; Revilla-Leon et al., 2017; Revilla-Leon et al., 2018a; Westover, 2019; Olea Vielba et al., 2020; Cucchi et al., 2021; Herschdorfer et al., 2021).

The accuracy of surgical implant placement is greatly improved by surgical guides constructed by 3D printing technology. Customized titanium mesh improves the accuracy of bone augmentation, and 3D printing technology can even be used to prepare personalized implants to meet individual patient needs (Revilla-León et al., 2020). During implant restoration, 3D printing technologies can replace certain manual operations, such as the manufacture of custom trays, working casts, etc., helping to reduce human error. Suitable tray and accurate dental models are key to restoration. There is evidence that 3D printed custom trays and models are sufficiently accurate, which significantly impacts the success of the final restoration. Additionally, implant-supported frameworks generated by CNC cutting are very popular, but this process results in a waste of materials, whereas implant frameworks fabricated by 3D printing effectively avoid this problem. The emergence of 3D printing has undeniably aroused the interest of many dentists and accelerated the clinical development of implantology. Some examples of the application of 3D printing technologies in dentistry are presented in Figure 2.

### 3.1 Applications of additive manufacturing in the surgical stage of implant treatment

#### 3.1.1 Surgical guides

**FIGURE 3:**
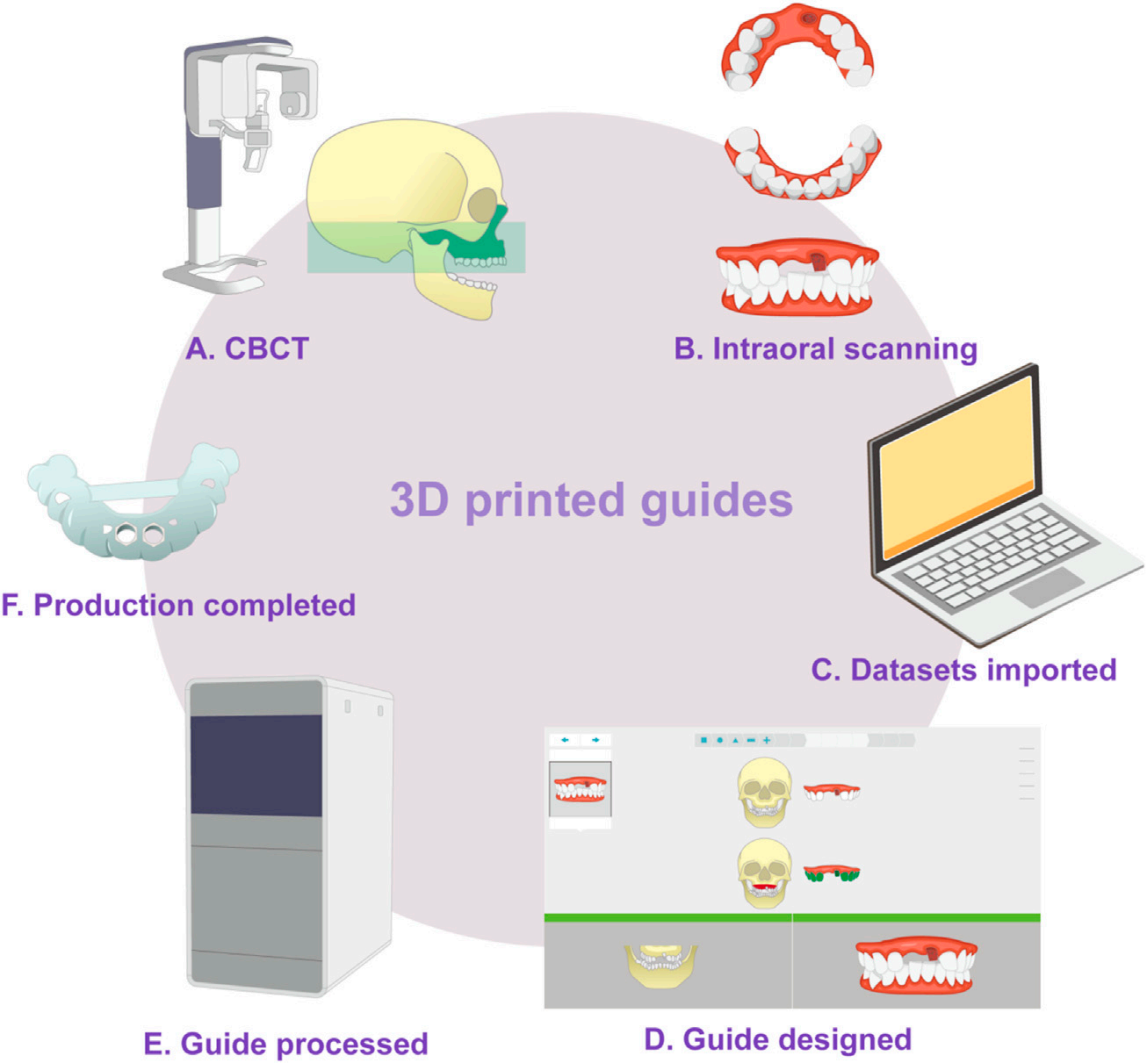
Schematic diagram of guide production. **(A)** Cone-beam computed tomography is used to generate 3D data from the teeth and jaws. **(B)** Three-dimensional information from the teeth and surrounding soft tissue is obtained by intraoral optical impression or scanned from a traditional plaster model. **(C)** In the guide design software, the two datasets are imported in turn and then matched, checked, and confirmed. **(D)** The guide is designed. **(E)** 3D printing is used to fabricate the guide. **(F)** The final guide is completed.

3D printed surgical guides have been used for more than 10 years, and the digital workflow of the guides is as follows. Virtual planning and design is conducted using computer software and digital workflows for planning and manufacturing, based on data obtained from 3D imaging, and these plans are then transmitted through 3D printed surgical guides (Rouzé L'Alzit et al., 2022; Unsal et al., 2020; Zhang et al., 2020). At present, SLA is the most widely used approach, due its economical nature and speed. Some newer technologies, such as PolyJet, can also be used to prepare guides (Henprasert et al., 2020). The guide manufacturing process is illustrated in Figure 3, taking tooth-supported guides as an example (Sedov et al., 2021).

The production of guides involves multiple, closely-linked steps, including data collection and integration, design of the implanting plan and guide structure, and manufacturing, and errors in previous steps will accumulate in the final guides (Putra et al., 2022; Rouzé L'Alzit et al., 2022). Factors influencing template accuracy can be roughly divided into three categories: 1) System error: that is, errors generated during CBCT scanning and data conversion, which cannot be controlled by humans; 2) Manufacturing error: related to the type of 3D printer (Zhang et al., 2022) (Table 1), selection of printing materials, use of supporting structures, slicing method and software types (Cho et al., 2021; Elliott et al., 2022; D. D; Rubayo, et al., 2021); and 3) Other factors.

**TABLE 1:** Accuracy of different 3D printing techniques for fabrication of surgical guides for use in dental implantology.

References	Printing techniques used	Main conclusions
Rouzé L'Alzit et al. (2022)	SLA, DLP, FDM, and SLS, Inkjet	1. Regardless of the 3D-printer technology used, small-extent surgical guides are more accurate than large-extent guides
		2. SLA and DLP produced similar results
		3. FDM was the least accurate
Henprasert et al. (2020)	SLA, PolyJet, and MultiJet	1. Planned and final implant positions are not influenced by the additive manufacturing technologies tested
		2. The additive manufacturing technologies tested allowed for accurate implant placement
Chen et al. (2019)	SLA, PolyJet, and DMP	1. PolyJet 3D printing is more accurate and reproducible than SLA 3D printing
		2. Printed Co-Cr metal surgical templates produced using the DMP 3D printer retain their initial accuracy and reproducibility after 1 month of storage
Gjelvold et al. (2019)	SLA and DLP	1. The tested desktop 3D printers can produce surgical guides with similar deviations to those generated by definitive implant position
		2. DLP printing was more accurate concerning deviations at the entry point and vertical implant position
Abduo and Lau, (2020)	DLP and FFF	1. Although the two printers generally have similar accuracy, the guides produced by DLP printers are more accurate than those generated by FFF.
Sommacal et al. (2018)	FFF and DLP	1. There is a statistically significant difference between templates printed with a professional DLP printer and those printed with a consumer FFF3D printer
		2. Consumer FFF 3D printers are not suitable for creating templates for implant-guided surgery
Pieralli et al. (2020)	SLA and FDM	1. The accuracy of surgical guides made by FDM is similar to SLA.
Sun et al. (2019)	SLA and FDM	1. Using an FDM-printed surgical template for implant implantation is as accurate as using an SLA template for single dental space indications
Koch et al. (2019)	SLA, MultiJet and PolyJet	1. The SLA guides have the smallest deviation, followed by PolyJet and MultiJet.
		2. The average 3D deviation of two printers of the same brand and model is significantly different
Sun Y et al. (2022)	SLA and FDM	1. The placement accuracy of the FDM guide is the same as that of the SLA guide for single posterior edentulous spaces
Wegmüller et al. (2021)	MJ, SLA, FFF, and DLP	1. The accuracy of MJ guides is higher than that of FFF and DLP guides (*p* < 0.01)

DLP, digital light processing; FDM, fused deposition modeling; FFF, fused filament fabrication; SLA, stereolithography; SLS, selective laser sintering; MJ, Material Jetting.

To reduce surgical complications caused by problems with 3D printed surgical guide production, it is vital to understand the limitations of 3D printing technology. Research has demonstrated that 50 µm layer printing provides better overall guide dimensions than 100 µm layer printing (Dalal et al., 2020). Further, printing angulation can influence the intaglio surface, as well as tube deviations (Dalal et al., 2020). Rubayo et al. showed that 0- and 45-degree build angles produced the most accurate surgical templates, while a 90-degree build angle generated the least accurate surgical templates (D. D. Rubayo, et al., 2021). Tahir et al. evaluated the effect of different printing directions on the placement accuracy of implant surgical templates made by DLP. The results showed that the horizontally printed templates showed excellent accuracy (Tahir and Abduo, 2022).

Regarding other factors that influence template accuracy, Zhou et al. (Zhou et al., 2018) conducted a comprehensive comparison of various clinical factors and concluded that guide accuracy may be affected by guide position (maxilla or mandible), guide fixation (fixation screw or not) (Pessoa et al., 2022), type of guide (total or partial) (Mangano et al., 2018; Lou et al., 2021; Fotopoulos et al., 2022; Gargallo-Albiol et al., 2022), flap approach (open flap or flapless), differences in implant system (Zhu et al., 2021), high temperature sterilization (Marei et al., 2019; Kirschner et al., 2022) and support mode (tooth-supported, mucosa-supported or mixed-supported) (Pan et al., 2022). In addition, Henprasert et al. concluded that there was no significant difference in accuracy between 3D-printed and milled guides, but found that the former had the advantages of high efficiency and reduced material waste (Henprasert et al., 2020). Mukai et al. compared the repeatability and accuracy of two surgical guides obtained using 3D printing and milling methods (Mukai et al., 2021) by overlaying images and the results revealed no significant differences in average mismatch between the two groups in terms of trueness (*p* = 0.529) or precision (*p* = 0.3021), indicating that both milling and printing manufacturing methods are suitable for guided surgery.

#### 3.1.2 Customized titanium mesh

**FIGURE 4:**
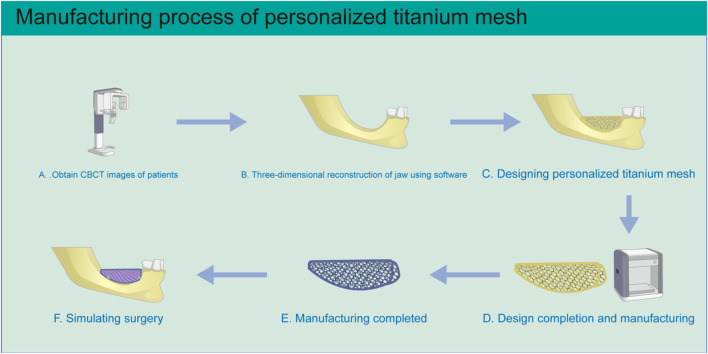
Schematic diagram of personalized titanium mesh production. **(A)** Three-dimensional CBCT image data of the patient’s jaws is obtained. **(B)** The three-dimensional jaw structure is reconstructed. **(C)** Personalized titanium mesh is designed according to the condition of alveolar bone defect. **(D)** After the design is completed, the file is imported into the 3D printer for manufacturing. **(E)** After printing is completed, the titanium mesh is treated by ultrasonic cleaning, sandblasting, and acid etching. **(F)** The situation is simulated during the operation.

The development of customized titanium mesh generated by 3D printing technology, with the aim of solving the shortcomings of traditional titanium mesh, has become a focus in GBR research and application (Xie et al., 2020). Based on patient CBCT three-dimensional jaw data, the ideal alveolar bone is virtually designed using CAD software, according to the shape of the dental arch and the expected implant position (Manzano Romero et al., 2021). Then, the matching customized titanium mesh is designed directly on the reconstructed alveolar bone model. Finally, customized titanium mesh is manufactured using SLM or EBM (Figure 4) (Xie et al., 2020; Cucchi et al., 2019; Seiler et al., 2018).

There are many advantages of 3D printed customized titanium mesh (Seiler et al., 2018; Hartmann et al., 2019), as follows.

• The cell structure, pore size, and thickness of 3D printed titanium mesh can be adjusted according to requirements (Jung et al., 2014).
• The scope of 3D printed titanium mesh is limited to the area of the bone defect rather than extending into areas with sufficient bone mass, avoiding overstretching of soft tissue. In the second stage of surgery, the doctor only needs to remove the titanium mesh through the alveolar crest incision, avoiding the need to turn the flap again.
• The use of customized titanium mesh can reduce the operation time, thus shortening exposure to general anesthesia, decreasing blood loss, decreasing wound exposure time, and simplifying the surgical procedure.
• Personalized titanium mesh has lower exposure rates, as confirmed by a meta-analysis conducted by Zhou et al. (Zhou et al., 2021). Titanium mesh avoids nerves and blood vessels in the initial design, which is of great significance in improving the accuracy of GBR.

Guidelines for designing 3D printed customized titanium meshes have been published (Sagheb et al., 2017; Hartmann and Seiler, 2020; Li L et al., 2021) and include.

• The bone mass around the implant, the shape of alveolar bone, and the condition of soft tissues are factors that should be considered in the design.
• The distance between the boundary of the titanium mesh and adjacent teeth, nerves, blood vessels and other important structures should be ≥2 mm.
• The position and number of nail holes should be determined according to the retention needs. Nail hole diameter can be designed according to that of the titanium nail to be used.

In terms of the accuracy of 3D printed titanium mesh, Sumida et al. (Sumida et al., 2015) confirmed that the maximum error between customized titanium mesh and CAD data is <300 μm, indicating that it has sufficient accuracy for GBR. Regarding mechanical properties, the average tensile strength and test stress of titanium mesh with a thickness of 0.5 mm and a pore size of 1 mm printed by SLM are 627 ± 41 and 541 ± 26 MPa, respectively, while the average elongation strength and micro-Vickers hardness are 7.2% ± 2.8% and 203 ± 5 HV, respectively, demonstrating that titanium mesh has good mechanical properties. Otawa et al. (Otawa et al., 2015) tested the accuracy of SLM titanium mesh. The results show that dimensional accuracy, pore structure accuracy and the error between CAD design and the scanned real product by overlapped images are tolerable, and the maximum error and average error are 292 μm and 139 μm, respectively.

In term of indications, customized CAD/CAM titanium mesh can be used in bone augmentation surgery for horizontal and vertical bone defects, particularly for cases with large area, complex, combined horizontal and vertical bone defects. Some clinical studies have focused on the bone augmentation effect of CAD/CAM titanium mesh as shown in Table 2. It is worth noting that many clinical studies have shown that customized titanium mesh cannot completely avoid the problem of titanium mesh exposure, but exposure does not necessarily affect the final bone augmentation effect (Zhou et al., 2021; Ciocca et al., 2018a; Hartmann and Seiler, 2020). From the perspective of design, some scholars have demonstrated this by optimizing the design of customized titanium mesh thickness and pore size and other parameters and making the shape of customized titanium mesh smooth, so as to reduce the possibility of exposure. In addition, some scholars suggest using fibrin rich in platelets or collagen membrane to cover titanium mesh to reduce the exposure rate (Cucchi et al., 2021; Sagheb et al., 2017).

**TABLE 2:** Different studies used 3D printing titanium mesh to obtain the effect of bone augmentation.

References	Bone graft material	Design of customized titanium mesh	Printing techniques used	Whether to use other barrier membranes	Main conclusions
Sagheb et al. (2017)	Particulate autogenous bone mixed with deproteinized bovine bone mineral	-	-	A resorbable collagen membrane or a resorbable collagen membrane, followed by platelet-rich fibrin (PRF) membranes	Six months after operation, CBCT showed an average increase of 6.5 ± 1.7 mm in vertical bone mass and 5.5 ± 1.9 mm in the horizontal direction, compared with that before surgery
Ciocca et al. (2018a)	Autologous bone and anorganic bovine bone in a 1:1 ratio	The mesh was calibrated at a 0.3-mm thickness, and holes in the mesh were calibrated at 1-mm diameter	DMLS	No	Six to 8 months after surgery, cone beam CT showed an average increase of 1.72–4.1 mm (average 3.83 mm) in the mandibular arch and 2.14–6.88 mm (average 3.95 mm) in the maxilla
Cucchi et al. (2021)	A mix 50:50 of autogenous bone and bone xenograft	The meshes were usually less than 0.5 mm in thickness	SLM	Patients requiring bone augmentation procedures were randomly divided into two groups: group A received only custom-made meshes (Mesh-) and group B received custom-made meshes with collagen membrane (Mesh+)	Although group B had superior outcomes to group A in regenerated bone volume, the use of custom-made meshes alone did not seem to be inferior to custom-made meshes covered with cross-linked collagen membrane, in terms of healing complication and regeneration rates
Li L et al. (2021)	Particulate autogenous bone chips, deproteinized bovine bone mineral, and platelet-rich-fibrin (i-PRF)	The thickness of the model is 0.3 mm, and the aperture is 2.0 mm. The edge of the mesh should avoid damage to the adjacent teeth, nerves, blood vessels and other important structures, and stay away from these structures at least 2 mm	DMLS	Dual layer of resorbable collagen membrane and concentrated growth factor matrix	According to the post-implantation CBCT evaluation, the patient-based average vertical bone gain was 3.55 ± 3.74 mm, and the horizontal average bone gain at 0, 2, and 4 mm below the implant platform was 4.06 ± 2.37, 5.58 ± 2.65, 5.26 ± 2.33 mm, respectively
Cucchi et al. (2020)	Autogenous bone and bone xenograft	The meshes were usually less than 0.5 mm in thickness	SLM	No	Measurements showed an average VBG of 4.5 ± 1.8 mm at surgical re-entry. Surgical and healing complications occurred in 30% and 10% of cases, respectively. Mean values of PBV, LBV, and RBV were 984, 92, and 892 mm^3^, respectively. The average RR achieved was 89%

VBG, vertical bone gain; PBV, planned bone volume; LBV, lacking bone volume; RBV, regenerated bone volume; RR, average regeneration rate.

In fact, there is another way AM can help GBR, as used by Li et al. (Li S et al., 2021). By collecting intraoral scanning and DICOM (digital imaging and communications in medicine) data from patients, the implant position can be digitally designed, and the alveolar bone digitally augmented around the ideal implant position. This process provides superior precision and efficiency relative to traditional GBR procedures, as reflected in the preoperative virtual bone augmentation design, with an increment of 0.5 mm beyond the contour of the labial bone arch to compensate for possible bone resorption during bone healing. After 3D printing of the reconstructed alveolar bone model, the titanium mesh was trimmed and prefabricated on the alveolar bone model. The preformed personalized titanium mesh has lower technical sensitivity and better plasticity than 3D printed titanium mesh (Xie et al., 2020). Indeed, the customized titanium mesh made by both methods increases the possibility of customized bone regeneration (Seiler et al., 2018).

#### 3.1.3 Dental implants

Interconnected pore structures are conducive to the transport of nutrients and increase surface roughness, which facilitates new bone formation and osseointegration (Chen et al., 2020; Yu et al., 2020). Moreover, porous structures reduce implant stiffness and generate an implant elastic modulus similar to that of the human jaw, thereby reducing stress shielding effects and allowing implants to be retained and function in the jaw for long periods of time (Wally et al., 2019). Titanium implants with uniform micron-scale porous structure can be produced by 3D printing (Mangano et al., 2014b). SLM and EBM are typically used to prepare dental implants, and implant materials are mainly titanium and titanium alloys, although some scholars have attempted to use zirconia (Pillai et al., 2021). Further, some researchers have proposed the use of titanium for the root portion and zirconium in the abutment portion to form one-piece implants that can achieve optimal osseointegration and ideal soft tissue attachment (Westover, 2019).

Using the characteristics of the complex geometric components formed by 3D printing, implants simulating the natural root can be customized. Personalized implants can be completely consistent with the shape of the patient’s extraction socket. In immediate implant application, this approach can achieve high initial stability and reproduce the perfect gingival profile of natural teeth (Figliuzzi et al., 2012). In addition, personalized implants have similar stress conduction and distribution characteristics to natural teeth (Moin et al., 2013).

To obtain personalized implants, teeth and jaw data are collected by computed tomography or CBCT before surgery, and a three-dimensional model of the teeth reconstructed using software. Then, a CAD model of the implant is generated, exported to an STL file, and transferred to specialist reverse software. The surface of the model is smoothed to generate a regular surface, and then transferred to CAD software for abutment design. After completion, an STL file of the implant and abutment is obtained and imported into the printer for processing and manufacture (F. G. Mangano F. G. et al., 2013; Westover, 2019). Due to deviations in precision, some researchers have incremented implant CAD models by dimension percentages (0%, 5%, and 10%) for clinical applications (F. G. Mangano F. G. et al., 2013). Another technique involves laser scanning of the root to construct the final implant after extraction, and design of macro-retainers on the implant surface to increase its stability following placement (Dantas et al., 2021).

Another class of implants, referred to as patient-matched implants, are less personalized than 3D printed root-analogue implants; for example, some scholars have used 3D printing to generate narrow-diameter implants for patients with insufficient alveolar bone width (F. Mangano et al., 2013b). In addition, 3D printing is also used in the manufacture of non-customized dental implants (similar to current commercial implants). The Italian “Tixos” implant, which is similar to a traditional implant, is produced by DMLS technology and has a variety of sizes to choose from (Mangano et al., 2012a). Furthermore, implants produced by DMLS have high fatigue strength and good corrosion resistance (Yang et al., 2020). Gehrke et al. confirmed that the mean fracture strength of DMLS implants with diameter 3.5 mm and length 16 mm can reach >1200 N (Gehrke et al., 2018): However, for two-stage dental implants, it is difficult to achieve a tight connection between a 3D printed implant and the abutment due to a lack of surface accuracy, but a tight connection can be achieved by machining of the connection structure in the 3D printing implant platform (Tunchel et al., 2016). The predicted survival rates of 3D printed implants confirmed in clinical studies are presented in Table 3.

**TABLE 3:** Clinical studies of 3D printed titanium implant.

References	Manufacturing method	Material	Implant features	Clinical applications	Main findings
Mangano et al. (2013a)	DMLS	Ti-6Al-4V	Root-analogue implants	Root-analogue implants were implanted in the sockets and restored with a single crown for 15 patients	1. At 1-year follow-up, implant survival rate was 100%
					2. Mean DIB was 0.7 (±0.2) mm
Mangano, et al. (2012b)	DMLS	Ti-6Al-4V	Standard implants	Implants were inserted in the edentulous mandible for 24 patients	1. After a 1-year loading time, implant survival rate was 98.9%
					2. DIB was 0.28–0.30 mm (95% CI, 0.24–0.32)
Mangano et al. (2015)	DMLS	Ti-6Al-4V	Standard implants	Implants inserted in the mandible to support ball attachment-retained mandibular overdentures for 24 patients	1. After 4 years of loading, overall cumulative survival rate was 96.9%
					2. DIB values were 0.38–0.25 and 0.62–0.20 mm at 1- and 4-year follow-up examinations, respectively
Mangano et al. (2012b)	DLMF	Ti-6Al-4V	Standard implants	201 implants (106 maxilla, 95 mandible) were inserted in 62 patients	1. Overall implant survival rate was 99.5%
					2. Mean DIB was 0.4 ± 0.2 mm
Mangano et al. (2013b)	SLS	Ti-6Al-4V	Standard implants	Implants placed in the posterior jaw for 16 patients	1. At 2-year follow-up, implant survival rate was 100%
					2. Implant success rate was 94.6%
					3. DIB was 0.4 ± 0.3 mm
Mangano et al. (2014b)	DMLS	Ti-6Al-4V	Standard implants	Implants used to support bar-retained maxillary overdentures for 30 patients	1. 3-year implant survival rates were 97.4% (implant-based) and 92.9% (patient-based)
					2. Biological complication incidence rates were 3.5% (implant-based) and 7.1% (patient-based)
					3. The incidence of prosthetic complication was 17.8% (patient-based)
Tunchel et al. (2016)	DMLS	Ti-6Al-4V	Standard implants	Eighty-two patients (44 male, 38 female; age range 26–67 years) were enrolled. A total of 110 3DP/AM titanium dental implants (65 maxilla, 45 mandible) were installed: 75 in healed alveolar ridges and 35 in post-extraction sockets. Prosthetic restorations included 110 single crowns	1. After 3 years of loading, six implants failed, for an overall implant survival rate of 94.5%
					2. Among the 104 surviving implant-supported restorations, 6 showed complications and were therefore considered unsuccessful, for an implant-crown success rate of 94.3%
					3. Mean DIB values were 0.75 (±0.32) mm and 0.89 (±0.45) mm after 1 and 3 years of loading, respectively

DIB, distance from the implant shoulder to the first visible bone-to-implant contact; DMLS, direct metal laser sintering; SLS, selective laser sintering.

Poly ether ether ketone (PEEK) 3D printed implants have also attracted attention (Basgul et al., 2021b; Sun C et al., 2022). PEEK is an aromatic polymer with characteristics of corrosion resistance, high temperature resistance, non-cytotoxicity, X-ray transmission, chemical stability, and good biological safety (Najeeb et al., 2016; Panayotov et al., 2016; Basgul et al., 2021a). Compared with titanium alloy, the elastic coefficient of PEEK is relatively low and closer to that of human cortical bone, which helps to reduce stress shielding effects at the bone-implant interface, thereby minimizing implant loosening and peri-implant bone loss (Han et al., 2022). Although it has many advantages, the main challenge for PEEK as a dental implant material is that it is a bioinert material with low surface energy; Therefore, appropriate strategies should be developed to improve the biological activity of PEEK and realize its potential benefits (Jung et al., 2019; Basgul et al., 2021b).

FDM and SLS 3D printing technologies can be used to print PEEK implants, and the former is most commonly used (Schmidt et al., 2007; Baek et al., 2022; Basgul & Spece et al., 2021). The molecular structure of PEEK does not change at high temperature; thus, no toxic substances are produced in the printing process (Prechtel et al., 2020; Shilov et al., 2022). Further, 3D printed PEEK implants have many advantages, including free design, interconnected porous structures and specific surface topography (Shishkovsky et al., 2013; Torstrick et al., 2018; Yuan et al., 2018). Han et al. systematically analyzed the biological activity of PEEK implants printed by FDM, including surface roughness, wettability, cell adhesion, metabolic activity, and proliferation (Han et al., 2019a). They found that, compared with the sandblasted surface of traditional molded or milled PEEK implants, the special surface morphology and porous structure of 3D printed PEEK implants played an important role in stimulating bioactive potential.

In addition, to improve the bioactivity of 3D printed PEEK implants, Han et al. used plasma surface treatment technology to introduce Ar or O_2_ functional groups into the surface of 3D printed PEEK, which significantly improved surface hydrophilicity and changed surface morphology and roughness (Han et al., 2022). Plasma-treated PEEK induced adhesion, metabolic activity, proliferation, and osteogenic differentiation of SAOS-2 cells *in vitro*. Su et al. developed a sulfonation strategy to create uniform micropores on PEEK lattice scaffolds fabricated by FFF (Su et al., 2020). The suitable lattice structure sulfonation time was 30–45 s, and the mean size of formed micropores was 0.19 ± 0.07 μm. Compared with the untreated PEEK scaffold, the micropore structure on the FFF printed PEEK lattice scaffold significantly improved cell attachment, spreading, proliferation, and bone-specific differentiation of MC3T3-E1 cells. More importantly, the existence of micropores on the lattice scaffold promoted the attachment of new soft tissue to PEEK implants.

In addition to biocompatibility studies, other investigations have been dedicated to improving the mechanical strength of PEEK (Chen et al., 2022). Numerous factors can influence the biomechanical qualities of 3D printed PEEK implants, including the size/shape of the test sample, printing temperature, printing speed, layer thickness, component orientation, nozzle diameter, and raster angle (Arif et al., 2018; Basgul & Spece et al., 2021;Basgul & Thieringer et al., 2021; Prechtel et al., 2020; Moby et al., 2022). PEEK formed by FFF has sufficient tensile, bending, and fracture strength (Arif et al., 2018), but the fatigue properties of these implants require further evaluation (Fabris et al., 2022). Sonaye et al. studied the printing parameter set of PEEK implants produced by FFF technology (Sonaye et al., 2022) and showed that the best printing parameters for PEEK implants are: nozzle temperature, 450°C; bedplate temperature, 150°C; chamber temperature, 90°C; layer thickness, 0.1 mm; and printing speed, 30 mm/s. The use of optimized process parameters resulted in implants with excellent fatigue performance. Further, reinforcement of PEEK with various materials, such as glass fiber, carbon fiber, and silicate based bioceramics, improves its mechanical strength to a certain extent (Han et al., 2019b; Petersmann et al., 2020; Taymour et al., 2022).

In summary, the excellent overall properties of PEEK mean that it has considerable prospects for application as dental implant material. Combined with 3D printing, customized and graded porous PEEK implants can be manufactured quickly, resulting in implants with better performance than those generated by traditional manufacturing processes. However, 3D printing of PEEK implants is mostly at the laboratory research stage, and wide application of PEEK implants in the clinic requires more supportive evidence from basic research and clinical trials.

### 3.2 Applications of additive manufacturing in the restoration stage of implant therapy

#### 3.2.1 Custom trays

Three-dimensional printing technology has been applied to fabricate custom trays for implants (Kim et al., 2019b). Revilla-León et al. and Piedra et al. described an implant impression technique for a complete arch, digitally designing metal splint structured and custom trays, which were generated using DMLS and DLP technology, respectively (Revilla-León and Özcan, 2017; Piedra and Revilla-Leon, 2018). Good matching between the metal splint structure and the impression copings makes it simple to locate in the patient’s mouth. Compared with manual individual trays, 3D printed custom trays have many advantages: First, custom trays have sufficient extension range and more uniform 3D impression material space between the splinting structure and the custom tray (Sun et al., 2017), providing more accurate oral tissue records; and second, they reduce the time required to fix the impression rod using materials such as resin, shorten clinical operation duration, and avoid the inaccuracies in definitive models caused by resin polymerization shrinkage (Kim et al., 2019a; Revilla-Leon and Ozcan, 2019).

Research confirms that 3D printed custom trays are stronger than traditional custom trays (Liu et al., 2019), which can be attributed to the parameters set in the manufacturing process. With increasing printing layer thickness, the tensile bond strength of trays first increases and then decreases, reaching a peak at 0.4 mm thickness, and printing time decreases sharply. Although bending and tensile strength decrease, dimensional printing accuracy remains constant from 0.1 to 0.4 mm, and then decreases at 0.5 mm, demonstrating that moderate layer thickness provides the best performance for 3D printing of custom trays (Liu et al., 2021).

To meet clinical requirements, tray materials should have both sufficient rigidity and dimensional stability and provide sufficient retention of impression materials. Xu et al. evaluated the bonding strength between three 3D printed custom tray materials (SLA, DLP, and FFF) and three elastomeric impression/adhesive systems [vinyl siloxane ether (VSXE), vinyl polysiloxane (VPS), and polyether (PE)] using the peel test (Xu et al., 2020). The results showed that the three 3D printing tray materials have good chemical compatibility with adhesives such as VSXE, VPS, and PE, and that 3D printing tray materials can provide sufficient clinical bonding strength with elastic impression/adhesive systems; when severe impression removal resistance is detected, it is recommended to use both PLA and VPS.

The studies described above confirmed that 3D printed custom trays have sufficient strength, hardness, and bonding strength with impression materials. Hence, 3D printing implant impression techniques could provide alternatives to conventional impression techniques for implant restoration.

#### 3.2.2 Implant models

Materializing digital impressions is among the earliest applications of 3D printing in dentistry (Revilla-Leon and Ozcan, 2019). The definitive implant model must ensure accurate implant location and relationship with adjacent teeth. Compared with traditional plaster models, 3D printed models have the advantages of being lightweight, resistant to damage, and having high finish, good wear resistance, and avoiding the inaccurate position of the analogue, which may be caused by artificial fixation of the implant analogue on the impression (Monaco et al., 2018; Tian et al., 2021). Moreover, 3D printed models overcome the disadvantages of digital models, as the physical model facilitates simple evaluation of the occlusal condition and interproximal contact (Buda et al., 2018).

Printed model inaccuracy results from accumulation of distortions caused by the acquisition method, parameters determined by the design software, and the printing process (Tian et al., 2021), which may be affected by many factors, such as scanner selection and the digitization process, among others. Papaspyridakos et al. (Papaspyridakos et al., 2020) proposed that the deviation of a printed model for use in implant restoration should ideally be <100 μm and should not exceed 150 μm.

Maria et al. (Maria et al., 2021) measured the physical positions of implants in a master model and analogs in printed resin models using a coordinate measuring machine. Three analog implant systems for 3D printed resin models [Straumann (ST), Core3DCentres (CD) and Medentika (MD)] were tested. Mean 3D linear distortion for ST (−155.7 ± 60.6 μm), CD (124.9 ± 65.0 μm), and MD (−92.9 ± 48.0 μm) differed significantly (*p* < 0.01), confirming that the implant analog system has a significant effect on the accuracy of analogs in 3D printed models. Mean absolute angular distortion did not differ significantly between ST (0.57° ± 0.48°) and CD (0.41° ± 0.27°), while both differed significantly from MD (2.11° ± 1.14°). Print orientation had a significant effect on 3D linear distortion, but no discernible trend could be found. Michelinakis et al. pointed out that, when choosing a semi-digital method, the cumulative deviation of the model in the 3D printing process may lead to a deviation in the position of the implant analogue, which depends on the printing technique and materials used (Table 4) (Michelinakis et al., 2021). In addition, Yousef et al. (Yousef et al., 2021) suggested that restoration be conducted as soon as possible after the model is printed, as the 3D printed model will deform during long-term storage.

**TABLE 4:** Accuracy of different 3D printing techniques for fabrication of implant models for use in dental implantology.

References	Printing techniques used	Main conclusions
Olea Vielba et al. (2020)	MultiJet	1. There was no significant difference in x-, y-, and z-linear, or XZ angular discrepancy between the conventional and additive manufacturing groups
		2. The AM group had a significantly higher median YZ angular discrepancy than the CNV group (*p* = 0.007)
Buda et al. (2018)	PolyJet and SLA	1. Significant differences in accuracy among the implant analog cast fabrication systems
		2. The PolyJet industrial printing system was more accurate than the conventional gypsum implant analog cast
Revilla-Leon et al. (2018a)	MJP1, SLA, MJP2, DLP	1. Regardless of the cast system, *x*-axes showed more distortion (42.6 μm) than y- (34.6 μm) and z- (35.97 μm) axes
		2. Among additive manufacturing technologies, MJP2 presented the highest (64.3 ± 83.6 μm), and MJP1 (21.57 ± 16.3 μm) and DLP (27.07 ± 20.23 μm) the lowest distortion, which did not differ significantly from that of conventional dental stone (32.3 ± 22.73 μm)
Rungrojwittayakul et al. (2020)	CLIP and DLP	1. All 3D printed models generated using CLIP and DLP printers had clinically acceptable levels of trueness
		2. Models produced using the CLIP printer exhibited significantly greater trueness, relative to the reference model, although the difference was small
Kim et al. (2018)	SLA, DLP, FFF, PolyJet	1. The PolyJet and DLP techniques were more precise than the FFF and SLA methods, with PolyJet exhibiting the highest accuracy for 3D model printing
Hazeveld et al. (2014)	DLP, 3DP, MJP	1. All replicas were sufficiently accurate and could be used interchangeably with plaster models

3DP, 3-dimensional printing; CLIP, continuous liquid interface production; DLP, digital light processing; FFF, fused filament fabrication; SLA, stereolithography.

#### 3.2.3 Implant frameworks

CAD/CAM technologies are widely used to design and manufacture frameworks for implant-supported prostheses (Kapos and Evans, 2014). After completing CAD of implant-supported frameworks, the virtual design is transformed into a physical object by addition or subtraction manufacturing (Revilla-Leon et al., 2019b). Compared with traditional casting technology, subtractive technologies reduce some clinical steps and eliminate certain human errors, but result in waste of materials and may fail to reflect the finer details of frameworks (Svanborg et al., 2018). Metal AM processes, such as SLM or EBM, are also used to fabricate implant frameworks, and effectively reduce material waste (Barbin et al., 2020). The mechanical properties of 3D printed implant frameworks can be comparable to, or even better than, those of traditional casting (Revilla-Leon et al., 2021). In addition, the shape of retainers on frameworks can be freely designed to increase the adhesion between the framework and the resin material.

The accuracy of the fit between implant frameworks and the underlying structures is an extremely important factor in avoiding biological and technical complications (Svanborg et al., 2018), which affect the long-term clinical success of implant-supported restorations. In an *in vitro* study, Revilla-Leon et al. evaluated discrepancies in the manufacture of titanium frameworks for implant-supported complete-arch prostheses manufactured using SLM and EBM(Revilla-Leon et al., 2018a). First, titanium frameworks for implant-supported complete-arch prostheses were designed using dental software. Then, frameworks were fabricated using SLM or EBM technology. The manufactured titanium frameworks and framework STL files were fitted and superimposed using a coordinate measuring machine. The results showed that there were no significant differences between SLM and EBM in the *x* and *y*-axes of implant frameworks, while the *z*-axis varied. The 3D discrepancies of all comparisons ranged from 60 ± 18 μm to 69 ± 30 μm, and the differences were not statistically significant. The discrepancy in the *y*-axis was largest (37–56 μm), followed by the x (16–44 μm) and z (6–11 μm) axes. Revilla-Leon et al. also compared the discrepancy at the implant abutment-prosthesis interface of complete-arch cobalt-chromium implant frameworks fabricated by additive and subtractive technologies before and after ceramic veneering in a separate study (Revilla-Leon et al., 2021). No significant differences were detected between the CNC and AM groups, except that the AM group presented a significantly higher discrepancy on the *x*-axis compared with the CNC group. Abu and Onoral. (2021) fabricated 3-unit Co-Cr frameworks with three indirect (conventional technique, polymethyl methacrylate milling, SLA) and two direct (SLM and soft alloy milling) methods. The mean vertical marginal discrepancy value of the SLM group (74.2 ± 20.5 μm) was significantly lower than those of all other groups (*p* < 0.05), demonstrating superior fitting accuracy. Barbin et al. (2020) evaluated the influence of milling, SLM, and EBM on full-arch fixed dental prostheses (FAFDPs) manufacture, in terms of marginal FAFDP misfits, prosthetic screw stability, and strain and stress on implant-supported systems, among other parameters. Compared with SLM and EBM frameworks, milled frameworks had the highest average marginal match, but the deviation of the former was within a clinically acceptable range. Ceramic veneer had no significant effect on the average marginal misfit values of any manufacturing process, while spark erosion reduced mean marginal misfit values for SLM and EBM titanium frameworks. On screw stability analysis, milled frameworks showed higher mean screw-loosening values, but after chewing simulations, none of the frameworks exhibited screw loosening.

In a recent systematic review (Thakur et al., 2021), the marginal fit and accuracy of complete-arch implant-supported frameworks, implant-retained fixed partial dentures, single implant crowns, and interim implant-retained restorations fabricated using AM and subtractive manufacturing methods were compared. The results showed that there was no significant difference in the marginal fit of single implant crowns or complete-arch implant frameworks between the two fabrication methods. For implant-supported fixed partial dentures, AM was superior to subtractive milling, but both digital fabrication methods produced implant-supported superstructures with clinically acceptable marginal fit.

The characteristic superficial texture of the layer-by-layer buildup in AM technologies results in a rough metal surface. A strong correlation between the roughness values on mating surfaces and implant-prosthodontic discrepancy has been reported. To achieve acceptable implant prosthodontic discrepancy, some scholars have combined 3D printing technologies and subtractive processing, electropolishing, sandblasting, or other post-processing techniques (Revilla-Leon et al., 2019b; Revilla-Leon et al., 2021). Ciocca et al. (2018b) compared the trueness and precision of frameworks manufactured with an SLM/milling hybrid technique (SLM/m) and conventional milling. The maximum misfit for the milled group was 20–35 μm, while there was no significant difference between SLM and SLM/m, with errors of 8–16 μm and 9–22 μm, respectively. Moreover, irrespective of the manufacture method, the trueness of titanium framework misfit was affected by framework span.

## 4 Discussion

The focus of this review was to provide an overview of AM technologies commonly used in implant dentistry and their application in the surgical and restoration stages of implant therapy. In addition, the accuracy of different 3D printing techniques for fabrication of printed parts is summarized in Table 2 and Table 4. The growing interest in 3D printing technologies clearly shows their potential impact on the future of implant dentistry (Nikoyan and Patel, 2020). With their advantages of high material utilization, ability to form complex geometric structures, and production of personalized products, 3D printing technologies have become an alternative method to generate components from CAD files. The various materials used in implant dentistry require the application of different AM techniques. Metal materials, such as personalized titanium meshes and titanium dental implants, can be manufactured using DMLS/SLM/EBM, whereas polymer materials, such as surgical guides and implant models, are fabricated using SLA, DLP, and PolyJet techniques.

Resin material products (surgical guides and implant models, etc.) produced by VPP technology have been widely studied and applied; however, there are specific areas that warrant further investigation. In particular, the numbers of patients and observation time in clinical studies of personalized titanium mesh generated by 3D printing have been limited to date; hence, the therapeutic effects of this approach require further study and verification. In future research, to ensure mechanical strength, continued optimization of personalized titanium mesh parameters, such as thickness, pore diameter, and shape, is required. In addition, the effects of combining personalized titanium mesh with absorbable collagen membrane, concentrated growth factor membrane, or other bone augmentation techniques warrant further exploration. Furthermore, 3D printing to manufacture dental implants has only recently been introduced; thus, more research and clinical studies are needed to understand the long-term safety and clinical efficacy of 3D printed implants. Future work will include study of the mechanical properties and structural characteristics, as well as printing process optimization of 3D printed dental implants, to achieve improved accuracy and performance (Oliveira and Reis, 2019). Finally, although it may seem that all oral implant medical devices can be made using 3D printers, a single technology may not be able to meet all patient needs. For some applications, 3D printing technology needs to be combined with milling or other cutting/finishing techniques, to generate a product that can achieve the final application goal (de Oliveira Campos et al., 2020).
